# Identification Of Small Molecule TRABID Deubiquitinase Inhibitors By Computation-Based Virtual Screen

**DOI:** 10.1186/1472-6769-12-4

**Published:** 2012-05-14

**Authors:** Tong Shi, Ju Bao, Nick X Wang, Jie Zheng, Dianqing Wu

**Affiliations:** 1 Department of Pharmacology, Yale University School of Medicine, New Haven, CT 06510, USA; 2 Department of Structural Biology, St. Jude Children's Research Hospital, Memphis, TN 38105, USA

## Abstract

**Background:**

Wnt/β-catenin-mediated gene transcription plays important roles in a wide range of biological and pathophysiological processes including tumorigenesis where β-catenin-mediated transcription activity frequently elevates. TRABID, a deubiquitinase, was shown to have a positive Wnt/β-catenin-mediated gene transcription and hence holds a promise as a putative anti-cancer target.

**Results:**

In this study, we used a combination of structure based virtual screening and an in vitro deubiquitinase (DUB) assay to identify several small molecules that inhibit TRABID DUB activity. However, these inhibitors failed to show inhibitory effects on β-catenin-mediated gene transcription. In addition, expression of TRABID shRNAs, wildtype TRABID, or the DUB activity-deficient mutant showed little effects on β-catenin-mediated gene transcription.

**Conclusions:**

TRABID may not be a critical component in canonical Wnt/β-catenin signal transduction or that a minute amount of this protein is sufficient for its role in regulating Wnt activity.

## Background

The evolutionarily conserved Wnt signaling pathway regulates various developmental processes during embryogenesis and plays an important role for tissue homeostasis in adults. Wnt signaling pathway also plays an important role in tumorigenesis, particularly the formation of inherited and sporadic colorectal cancer as result of adenomatous polyposis coli (APC) mutation that leads to β-catenin accumulation in the nucleus [1-4]. Nuclear beta-catenin binds to and functions as a cofactor of lymphoid enhancer-binding factor (LEF-1) [5] and T cell factors (TCF) [6] to stimulate the transcription of Wnt target genes [7]. Wnt-β-catenin signaling is essential in sustaining the cancer stem cell phenotype and is also involved in the transformation into malignant human squamous cell carcinomas [8]. The loss of Wnt signaling pathway component APC in stem cells results in progressively growing neoplasia [9]. Activation of Wnt/TCF pathway is also a determinant of lung adenocarcinoma metastasis to brain and bone. The phenotype of those metastatic derivatives of adenocarcinoma resembles the bronchioalveolar stem cells [10]. Thus, it holds promises to prevent and/or treat cancers by targeting Wnt signaling pathway components.

TRAF-binding domain (TRABID), one of deubiquitination enzymes, was recently reported to specifically and positively regulate Wnt signaling pathway [11]. TRABID is composed of a TRAF-binding domain in the C-terminus and three Zinc-finger (ZnF) motifs at the N-terminus [12]. TRABID preferentially binds to lysine 63-linked polyubiquitin chains (K63 chains), but not K48-linked polyubiquitin chains (K48 chains), and specifically cleaves K63 chains [11]. K63-linked ubiquitination was suggested to regulate substrate activity rather than protein stability [13-16]. The catalytic residues of TRABID reside in the OTU (ovarian tumor) domain within the TRAF-binding domain [11].

The OTU domain is conserved within the members of the OTU family of DUBs that include A20 and possess the cysteine protease activity [17]. TRABID was also shown to bind to APC and may be responsible for its deubiquitination. However, the direct interaction between APC and TRABID was not detected [11]. Knockdown of TRABID with RNAi resulted in downregulated expression of canonical Wnt target genes and decreased Wnt transcription activity, whereas its knockdown does not affect TNF-2 pathway [11]. Epistasis analysis suggested that TRABID might act downstream of beta-catenin stabilization and affect the interaction of beta-catenin with LEF1 [11]. Furthermore, TRABID heterozygosity suppressed the rough eye phenotype caused by ectopic Wingless expression in fly, but did not affect the phenotypes caused by the inhibition of Notch signaling or inhibition of EGF signaling. These results suggest that TRABID might be a potential drug target for controlling Wnt pathway activation [11].

In this study, we screened for small molecule inhibitors of TRABID by employing a combination of structure based virtual screening and an in vitro DUB assay. We searched for compounds in a chemical library from the National Cancer Institute that potentially bind to TRABID catalytic site based on the crystal structure of A20 catalytic domain [17]. We identified several compounds that are able to inhibit the DUB activity of TRABID. However, these inhibitors failed to show inhibitory effects on Wnt activity. Furthermore, neither shRNAs that silenced TRABID efficiently nor overexpression of wild type (WT) TRABID or its DUB activity-deficient mutant showed inhibitory effects on canonical Wnt signaling activity.

## Results and discussion

**Figure 1:**
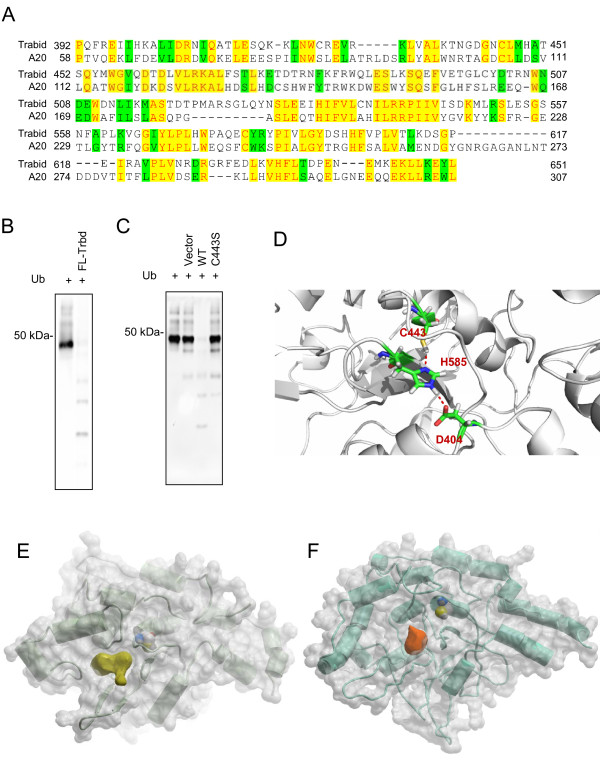
**TRABID DUB activity assays and structural modeling of TRABID OTU domain. A**) Sequence alignment of TRABID OTU domain and A20 OTU domain, the identical (yellow) and homologous (green) pairs are highlighted. B,C) Cleavage of K63 Chains by Recombinant TRABID. Hexa-K63 ubiquitin (0.01 ug/ul) were incubated with recombinant full length TRABID protein prepared from bacteria (**B**) or Flag-TRABID or Flag-TRABID-C443S precipitated by anti-FLAG agarose from transfected HEK293T cells (**C**) for 3 hrs in the DUB reaction buffer. Samples were analyzed by Western under a non-reducing condition using an anti-ubiquitin antibody. D-F) Modeled structure of TRABID OTU domain based on the crystal structure of A20 OTU domain. The modeled catalytic site of TRABID OTU domain is shown in **D**. The detected potential pocket of TRABID OTU domain is shown in E and the corresponding pocket in A20 is shown in F for comparison. The modeled Cys443 is highlighted in sphere representation in **E** and **F**.

To screen for compounds that can inhibit TRABID DUB activity in vitro, we established an assay to measure the DUB activity of TRABID. Consistent with the previous report by Tran et al. [11], we observed that recombinant TRABID proteins purified from an E coli expression system or pulled down from HEK293 cells overexpressing Flag-TRABID by an Flag antibody were able to specifically cleave hexa-K63 (Figure 1B,C), but not penta-K48 (data not shown), ubiquitin chains. We also tested a TRABID DUB-deficient mutant containing a substitution mutation (C443A), a residue located in the OTU domain and critical for its catalytic activity [11]. The mutation abrogated the ability of TRABID to cleave the Hexa-K63 ubiquitin substrate (Figure 1C), confirming the importance of this residue for the DUB activity. Homology modeling of TRABID OTU domain based on the crystal structure of A20 OTU domain [17] revealed that the common cysteine catalytic site was well conserved in TRABID. This site is characterized by a cysteine residue that forms an electrostatic network with a histidine and a residue with an acidic side chain. In TRABID, these 3 residues are C443, H596 and D410, respectively, based on the homology model (Figure 1D).

Searching on the surface of the equilibrated structure of TRABID OTU domain modeled from the crystal structure of A20 OTU revealed that there was a potential pocket adjacent to the catalytic center of OTU (Figure 1E). This pocket is part of a distal ubiquitin binding site at the catalytic site in A20 [17]. Because of its spatial proximity to the catalytic site and its role in ubiquitin binding, it is reasonable to postulate that targeting this site may block the entrance of the substrates and hence inhibit the OTU DUB activity. Of note, during the molecular dynamics refinement, considerable conformational changes occurred in this site, making it more expanded comparing with the pocket size in the A20 crystal structure (Figure 1F).

**Figure 2:**
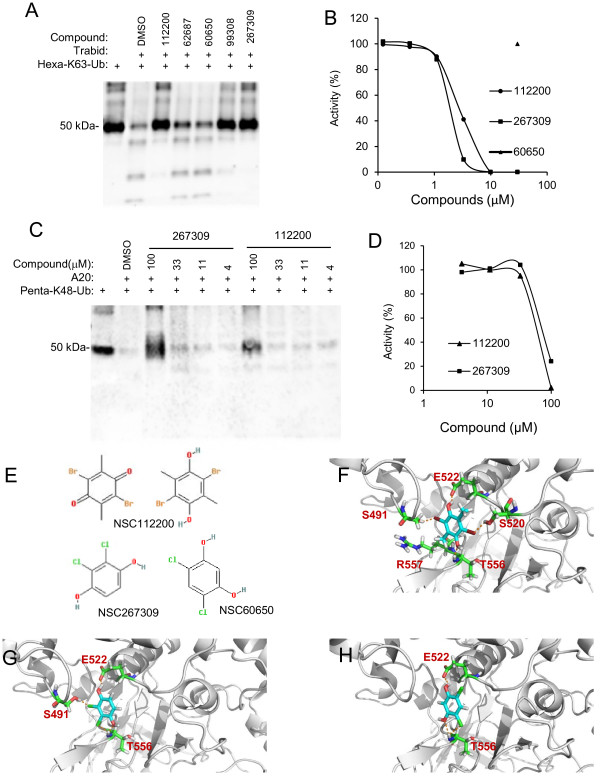
**Identification of TRABID DUB inhibitors. A,B**) Effects of compounds on TRABID DUB activity. Compounds (30 μM in A) were incubated with Hexa-K63 ubiquitin and immunoprecipitated FLAG-TRABID for 3 hrs. Samples were then analyzed by Western under a non-reducing condition using an antiubiquitin antibody. The DUB activity was quantified in B by determining the optical density of hexa-K48 ubiquitin bands at 50 kD. The band without TRABID was taken as 100% and the one with TRABID and DMSO as 0%. The experiments were repeated three times in duplicates, and errors are less than 5%. **C,D**) Effects of compounds on A20 DUB activity. Hexa-K63 ubiquitin was incubated with compounds and immunoprecipitated FLAG-A20 and analyzed by Western under a nonreducing condition using an anti-ubiquitin antibody. Quantification of DUB activity in D was carried out as described in **B**. **E**) Chemical structures of NSC112200, NSC267309 and NSC60650. **F**) Molecular modeling of the binding of NSC112200 to TRABID OTU **G**) Molecular modeling of the binding of NSC267309 to TRABID OTU **H**) Molecular modeling of the binding of NSC60650 to TRABID OTU.

**Figure 3:**
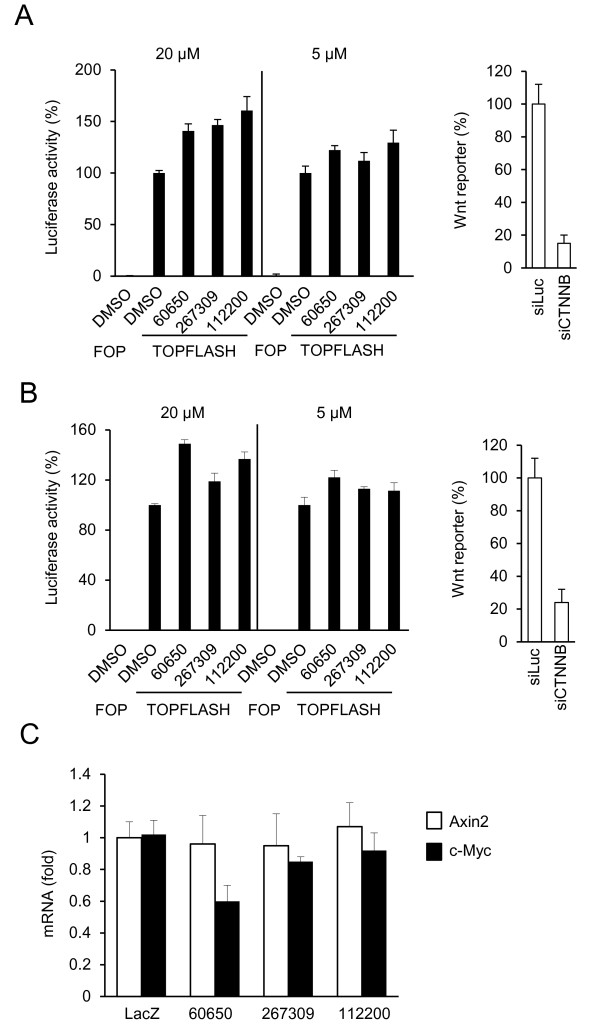
**Effect of TRABID inhibitors on Wnt/**β**-catenin-mediated transcriptional activity. A,B**) TRABID inhibitors fail to inhibit Wnt reporter gene activity. HCT-116 or SW480 cells were transfected with FOPFLASH or TOPFLASH and a plasmid expressing Renilla luciferase and incubated with the compounds for 24 hours. As a control the cells were also transfected with an β-catenin siRNA (siCTNNB). Normalized Wnt reporter gene activity is shown. The experiments were repeated three times in triplicates **C**) Effect of TRABID inhibitors on endogenous Wnt target gene expression in SW480 cells. SW480 cells were incubated with the compounds (20 μM) for 24 hours, and relative AXIN2 and c-Myc mRNA levels were determined by qRT-PCR normalized on the levels of β-actin mRNA. Experiments are performed in triplicates, and data are presented as means ± SD.

**Figure 4:**
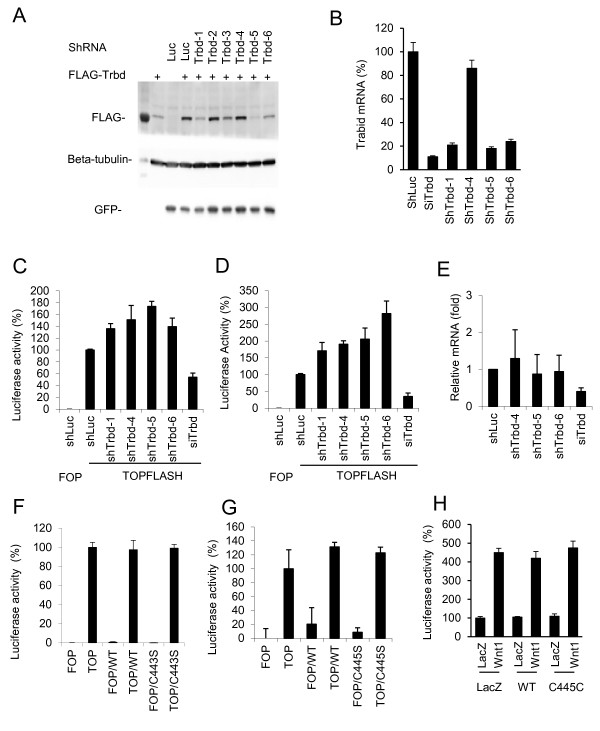
**Effects of TRABID shRNAs or overexpression on Wnt reporter gene activity and Axin2 expression. A**) Effects of TRABID shRNAs on TRABID expression. HEK293T cells were cotransfected with GFP (an internal control), FLAG-TRABID, and one of TRABID shRNA. Western analyzed was performed 48 hours after transfection. **B**) TRABID shRNA knockdown efficiency. HCT116 cells were transfected with shRNAs and sorted for YFP positive cells. As a control, HCT116 cells were transfected with the TRABID siRNA (siTrbd). Relative TRABID mRNA levels were determined by q-RT-PCR. **C,D**) Effects of TRABID shRNAs on Wnt reporter gene activity. HCT116 (B) or SW480 (C) cells were cotransfected with FOPFLASH or TOPFLASH, Renilla luciferase, and one of the TRABID shRNAs. TOPFLASH activity was detected 72 hrs after transfection. **E**) Effects of TRABID shRNAs on Axin2 expression in SW480 cells. Cells were transfected with one of the TRABID shRNAs for 72 hours, and total RNA was extracted. The levels of Axin2 mRNA were determined by qRT-PCR using beta-actin as control. **F,G**) Effect of overexpression of TRABID WT or C443S mutant on Wnt reporter gene activity. SW480 (E) or HCT116 (F) cells were cotransfected with WT TRABID or C443S TRABID and TOPFLASH or FOPFLASH. After 24 hrs, luciferase activity was determined. Experiments are performed in triplicates, and data are presented as means ± SD. **H**) Effect of expression of TRABID WT or C443S mutant on Wnt reporter gene activity in HEK293 cells. Cells were transfected as indicated, and luciferase activity was determined next day.

We performed hierarchical virtual screening towards this pocket as described in the “Materials and Methods”, and requested 200 compounds that were ranked highest in the virtual screen and were provided with 125 by NCI. These 125 compounds were screened for their abilities to inhibit the cleavage of Hexa-K63 ubiquitin by TRABID. Seventeen of these compounds showed more than 50 percent (denote with ++ in Additional file 1. Table S1) and five showed between 25–50 percent (denote with + in Additional file 1. Table S1) inhibition of TRABID DUB activity. Figure 2A shows a representative Western blot used in the TRABID DUB activity assay. All of the positive compounds were retested in a dose-dependent experiment. Figure 2B shows the data for the two of the strongest inhibitors, which showed the IC50 values of about 3 μM. Neither compound showed significant inhibition of A20 up to 30 μM, though both inhibited A20 at 100 μM (Figure 2C-D). Compound NSC60650 as well as a number of other compounds shown in Additional file 1. Table S1, which share similar chemical structures to NSC112200 and NSC267309 (Figure 2E), did not inhibit TRABID DUB activity (Figure 2B), suggesting that the two hydroxyl groups and their locations on the benzene ring may be important for the inhibitory activity. The molecular modeling of the bindings of NSC112200, NSC267309 and NSC60650 to TRABID OTU domain suggests that while all three compounds can form electrostatic interactions with the side chain of E522 and the backbone NH of T556, both NSC112200 and NSC267309 can form an additional hydrogen bond with the side chain of S491 (Figure 2F-H). Moreover, NSC112200 can form another hydrogen bond with the side chain of S520, and its one of methyl groups provides extra hydrophobic interaction with the carbon atoms located at the side chain of R557 (Figure 2F). The rescoring of binding free energies by ICM shows that the binding free energies of NSC112200 and NSC267309 with TRABID OTU are quite similar (−20.28 kcal/mol and −20.03 kcal/mol, respectively), while the binding free energy of NSC60650 with TRABID OTU is −16.89 kcal/mol, which is 3.39 kcal/mol higher than NSC112200. On the other hand, the calculated binding free energies of NSC112200 and NSC267309 with A20 OTU are −15.23 kcal/mol and −13.43 kcal/mol, respectively. As the high binding free energy means a lower binding affinity, our modeling predictions are consistent with our experimental data. TRABID was shown to play a positive role in canonical Wnt signaling, and its DUB activity appeared to be essential for this function [11]. We hence tested the TRABID DUB inhibiting compounds for their effects on canonical Wnt signaling. SW480 or HCT116 colorectal cancer cell lines, which were used in the previous study for TRABID’s role in Wnt signaling [11], were transfected with a Wnt reporter gene construct TOPFLASH and incubated with compounds for 24 hrs. Because these two cell lines contain mutations in the APC and β-catenin genes, respectively, there was high reporter gene activity in these cells (Figure 3A-B). However, neither NSC112200 nor NSC267309 inhibited the reporter gene activity (Figure 3A-B). As controls knockdown β-catenin resulted in significant reduction in Wnt reporter gene activity (Figure 3A,B). We also tested other TRABID inhibitors in Additional file 1. Table S1, and none of them inhibited the reporter gene activity (data not shown). We then examined two endogenous Wnt target gene expression. Neither Axin2 nor c-Myc expression in SW480 cells was affected by NSC112200 or NSC267309 treatment (Figure 3C). These results together suggest that TRABID might not be involved in Wnt$β-catenin-mediated gene transcription regulation. To further evaluate the role of TRABID in the regulation of Wnt/β-catenin-mediated gene transcription, we generated 6 constructs that produce six independent shRNAs for TRABID. When these shRNAs were coexpressed with TRABID cDNA in HEK293 cells, they showed varying efficiencies in suppressing TRABID expression with shTrbd5 and 1 being the most efficiency and shTrbd2 and 4 showing little effects (Figure 4A). We also evaluated their knockdown efficiencies using quantitative RT-PCR analysis of RNAs from YFP-positive HCT116 transfectants (shRNA and YFP were in the same transcript) (Figure 4B). However, expression of these shRNAs had no inhibitory effect on Wnt reporter gene expression in either SW480 or HCT-116 cells (Figure 4C,D). These shRNAs also did not show inhibitory effect on the expression of endogenous Wnt target gene Axin2 in SW480 cells (Figure 4E). Furthermore, overexpression of wildtype TRABID or its DUB-deficient mutant had little effects on Wnt reporter gene expression in either SW480 or HCT-116 cells (Figure 4F,G) or in HEK293 cells (Figure 4H). Thus, we failed to demonstrate that TRABID has an important role in Wnt target gene expression regulation in these two cancer cells.

**Figure 5:**
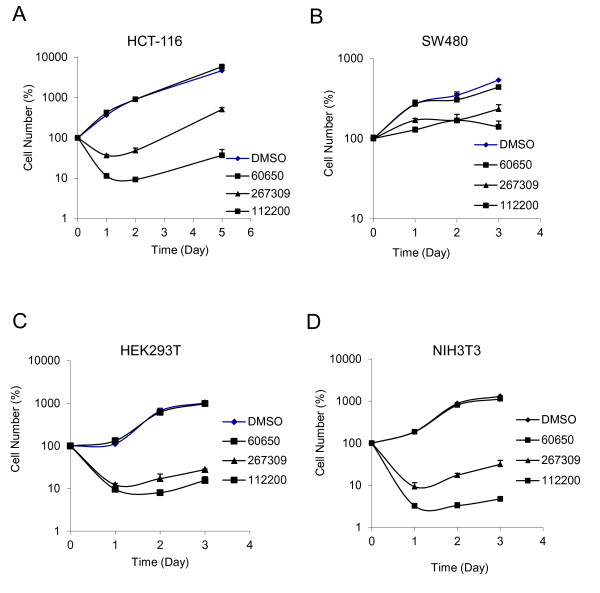
**Effect of TRABID inhibitors on cell growth. ** Compounds (20 μM) were added to cells at seeding and cell growth was determined by counting live cells at indicated times. Experiments are performed in triplicates, and data are presented as means ± SD.

Nevertheless, both TRABID inhibitors NSC112200 and NSC267309, but not the non-active analog NSC60650, inhibited the growth of not only the colorectal tumor cell lines HCT-116 and SW480, but also two para-normal cell lines NIH3T3 and HEK293 at 20 μM (Figure 5). These compounds showed no effects on the growth of these cells at 5 μM (data not shown). Because of the lack of effect of Compound NSC60650 on cell growth, we believe that the effects of Compounds NSC112200 and NSC267309 are likely due to their inhibition of the deubiquitinase activity.

Wnt signaling plays a crucial role in cancer cell growth and cancer stem cell maintenance, which are believe to be responsible for cancer metastasis and poor prognosis of chemotherapy. Thereby, regulation of Wnt signaling holds promise in chemotherapy of cancer. TRABID, one of K63-specific DUB enzymes, became an attractive candidate to achieve this task since it has been reported to regulate Wnt signal positively [11], as inhibition of TRABID presumably decreases Wnt signaling activity. However, in this study, we failed to confirm the notion that TRABID positively regulates canonical Wnt signaling based on several lines of evidence. Firstly, the compounds, which blocked TRABID DUB activity in vitro, failed to inhibit Wnt transcription activity in two tumor cell lines whereβcatenin-mediated transcription are known to be elevated. Secondly, the TRABID shRNAs failed to downregulate Wnt/β-catenin-mediated transcription activity or endogenous Wnt target gene Axin2 mRNA levels in these tumor cells. Thirdly, overexpression of WT and C443S mutant TRABID failed to show any effect on Wnt target transcription activity. We did observe that the siRNA described in the previous report [11] was able to inhibit Wnt reporter gene activity (Figure 4C-E). One possibility is that the effect of the siRNA reported in [11] is off target. A less likely possibility is that a minute amount of TRABID is sufficient for its regulation of Wnt signaling in these cells, and our shRNAs or inhibitors were not efficient enough to alter Wnt activity. Nevertheless, the fly data [11] have already suggested that TRABID might not be a core component of Wnt signaling. The observation that the TRABID inhibitors were able to inhibit cell growth suggests that TRABID may have a role in cell growth regulation. However, the mechanisms for this role of TRABID need to be further investigated.

## Conclusions

We have used computation-based virtual screen to identify chemical inhibitors for TRABID, but failed to confirm that TRABID has a significant role in Wnt signaling. Nevertheless, inhibition of TRABID may inhibit cell growth.

## Methods

Construction of TRABID expression vectors and shRNA expression vectors Vectors expressing GST-fused full length (FL) and N-terminal fragment (NT) (amino acides: 1–354) of human TRABID were constructed using pET-GST vector (EMD Chemicals Inc., Gibbstown, NJ). Proteins were expressed in bacteria and purified using glutathione sepharose 4B (Roche Diagnostics, Indianapolis, IN). FLAG-expression vectors for expression of the full-length and DUB-deficient (C443S) TRABID in mammalian cells were gifts from Dr. Hoanh Tran. FLAG-tagged proteins were immunoprecipitated using the M2 agarose affinity gel (Sigma-Aldrich, St. Louis, MO). TRABID DNA sequences were confirmed by DNA sequencing. TRABID shRNA targeting sequences were selected using the RNAi Codex algorithm (http://katahdin.cshl.org:9331_portal_scripts_main2.pl) [18] and the shRNAs were expressed using Migr-CMV-YFP-miR30 vector as previously reported [19]. The shRNA target sequences are: shTrabd1, TGTCTCAACAAGCAGCAAAGT; shTrabd2, AGGAGCTAGGTAATGAGGAAC; shTrabd3, TGTCAGAACGTGGAATTAAGT; shTrabd4, TGATCATCCCAGACCTAATAA; shTrabd5, CTGGCACATATTCTTAGACGA; shTrabd6, TTGGAAAAGTCCGATTGCTCT. β-catenin siRNA [20] and TRABID siRNA [11] target sequence were described previously.

### DUB enzymatic activity assay

For determining TRABID DUB enzymatic activity, hexa-K63 ubiquitin (0.01 ug/ul, Boston Biochem Inc., Cambridge, MA) and the compounds were incubated with TRABID immunoprecipitated from transfected HEK 293 T cells or prepared from bacteria for 3 hrs at 37°C in a DUB reaction buffer (50 mM HEPES at pH 7.4, 150 mM KCl, 10 mM DTT, 5% glycerol, 0.01% Triton X-100) [11]. Samples were applied to SDS-PAGE under non-reducing conditions and then transferred onto members. Ubiquitin chains were detected using a rabbit anti-ubiquitin antibody (Bethyl Laboratories Inc., Montgomery, TX).For determining A20 DUB enzymatic activity, Penta-K48 ubiquitin (0.04 ug/ul, Boston Biochem Inc., Cambridge, MA), 1.5 uM of A20 catalytic domain (Boston Biochem Inc., Cambridge, MA), and various concentrations of compounds were incubated for 3 hrs at 37 C in an A20 DUB reaction buffer (50 mM HEPES at pH 8.0, 3 mM DTT, 0.01% Brij-35) [21].

Homology modeling and potential pocket identification There are two crystal structures of A20 OTU domains deposited in PDB with nearly identical sequences and structures. Because The OTU domain of A20, shared a high degree of amino acid homology with that of TRABID, its sturcutre is the ideal template for the homology modeling of TRABID OTU structure. We used one of the A20 OTU structures (from PDB ID 2VFJ [17]) as the template for homology modeling.. The homology model of TRABID was built by the Prime module of the Schrodinger molecular modeling package [22,23]. A 3 ns molecular dynamics was performed by AMBER10 [24] to equilibrate the initial model since there is considerable gap (15%) between the sequences of TRABID and A20 (Figure 1A). The final equilibrated structure is used for the further modeling. The ICM PocketFinder module based on an algorithm of utilizing a Van de Waals grid potential map and a carbon probe [25] was used to detect potential relevant pockets around the catalytic cysteine residue of the TRABID OTU domain.

### Virtual screening

Since there is no any knowledge about the characteristics and properties of the potential ligands, it is necessary to search all the compounds in the library to find potential hits. We thus carried out virtual screens of the National Cancer Institute (NCI) database (http://129.43.27.140/ncidb2) for chemical compounds that could potentially bind to the detected cavity. This database includes the coordinates of 250,251 small (M.W. < 1,000 Da) chemical compounds. In order to eliminate the large amount of irrelevant compounds we used Glide module [26,27] in the Schrodinger package to perform hierarchical searches. The scoring function of High-throughput in Glide, which is less accurate but very fast, was used to perform the first round screening. Subsequently, the top 5000 compounds were selected and further tested with the normal scoring function in Glide. Finally, the most precise, but slower, scoring function in Glide [28] was used to perform the final screening with the top 500 compounds, and the top 200 compounds were requested from NCI and evaluated by the experimental assay.

In order to further interpret the inhibitory mechanism, the binding free energies of two strong TRABID deubiquitinase inhibitors, NSC112200 and NSC267309, identified in the screen were reevaluated by using the ICM empirical function, which is fast and fairly accurate in predicting relative binding free energies [29]. The binding free energy of NSC60650, which shares similar chemical structure with NSC112200 and NSC267309 but shows no inhibition, was also recalculated for comparison. The overall complexes were optimized and the binding free were calculated using Equation:

(1) $$\Delta {\text{G}}_{\text{binding}}=\Delta {\text{G}}_{\text{vw}}+\Delta {\text{G}}_{\text{hb}}+\Delta {\text{G}}_{\text{to}}+\Delta {\text{G}}_{\text{el}}+\Delta {\text{G}}_{\text{sf}}$$

Where ∆G_vw_ is the Van der Waals energy, ∆G_hb_ is the htdrogen bonding energy, ∆G_to_ is torsional energy, ∆G_el_ is electrostastic energy; and ∆_sf_ is the surface energy.

### TOPFLASH reporter assays

For testing the effect of compounds, cells were seeded in 48-well culture plates at a density of 0.2-0.3 million/ml, 250 ul/well and transfected with TOPFLASH or FOPFLASH and Renilla luciferase using the Lipofectamine Plus reagent (Invitrogen Corporation, Carlsbad, CA). The compounds were added 3 hrs later. For shRNA knockdown experiments, cells were transfected with an shRNA expressing plasmid and re-transfected with the Wnt reporter gene plasmids and the shRNA two days later. Wnt reporter gene activity was determined 24 hrs after the re-transfection and presented after normalization against the Renilla luciferase activity.

### Quantitative RT-PCR

Cells were plated into 6 well plates at a density of 0.2 million/ml for 12–16 hrs. For compound inhibition experiments, cells were incubated with 20 μM of a compound for 24 hrs. For shRNA inhibition experiments, cells were transfected with the shRNAs and incubated for 72 hrs. Total RNAs were extracted using the RNAeasy mini kit (Qiagen, Valencia, CA) and transcripted into cDNAs. The levels of Wnt target gene mRNAs were determined using a MyiQ Single color real-time PCR detection system (Bio-Rad, Hercules, CA) with beta-actin as a control.

### Cell growth assay

Cells were seeded at a density of 0.05 million/ml in 24-well culture plates and added with 20 μM compound. On indicated date, the numbers of living cells were counted using Guava Easycyte Mini Flow Cytometry System (Millipore Corporation, Billerica, MA).

## Authors’ contributions

T. S. carried out biological experiments and drafted the manuscript. J.B., N.X.W. and J.Z. performed the virtual screen, modeling and manuscript preparation. D.W. participated in experimental design and drafted the manuscript. All authors read and approved the final manuscript

## Supplementary Material

Additional file 1 **Table S1. **TRABID inhibitory activity and structures of additional compounds. Click here for file
