# Supplementary figures and images for: Identification Of Small Molecule TRABID Deubiquitinase Inhibitors By Computation-Based Virtual Screen
Source: BMC Chem Biol. 2012 May 14;12:4. doi: 10.1186/1472-6769-12-4 (PMC3475094; doi:10.1186/1472-6769-12-4)

| NCI Compound NO | Structure                                                                           | Inhibition | IC50 (uM) |
|-----------------|-------------------------------------------------------------------------------------|------------|-----------|
| 29027           | 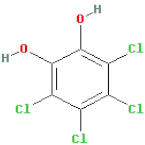   | ++         | 2.532     |
| 10858           | 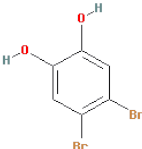   | ++         | 2.84      |
| 267309          | 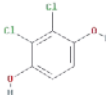   | ++         | 2.471     |
| 112200          | 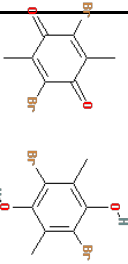 | ++         | 3.79      |
| 48667           | 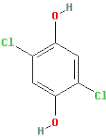 | ++         | 3.805     |
| 427             | 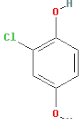 | ++         | 3.887     |

|        |                                                                                                                                                                         |    |       |
|--------|-------------------------------------------------------------------------------------------------------------------------------------------------------------------------|----|-------|
| 36936  | 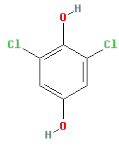                                                                                       | ++ | 4.517 |
| 133363 | 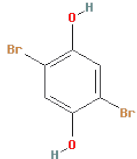                                                                                       | ++ | 7.167 |
| 29034  | 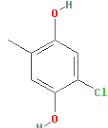                                                                                       | ++ | 7.54  |
| 226243 | 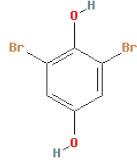                                                                                     | ++ | 7.556 |
| 4858   | 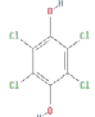 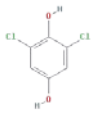 | ++ | 9.847 |
| 508878 | 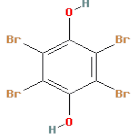                                                                                     | ++ | 14.98 |

|        |                                                                                                                                                                        |    |       |
|--------|------------------------------------------------------------------------------------------------------------------------------------------------------------------------|----|-------|
| 168515 | 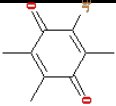<br>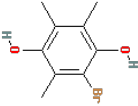 | ++ | 15    |
| 36929  | 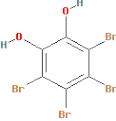                                                                                      | ++ | 15.26 |
| 3977   | 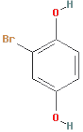                                                                                      | ++ | 16.38 |
| 36657  | 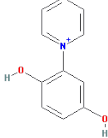                                                                                    | ++ | 33.86 |
| 5934   | 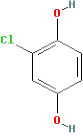                                                                                    | ++ | 35.75 |
| 99300  | 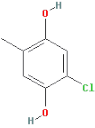                                                                                    | +  |       |

|       |                                                                                     |   |  |
|-------|-------------------------------------------------------------------------------------|---|--|
| 48686 | 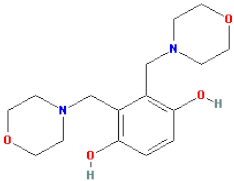   | + |  |
| 5718  | 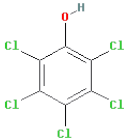   | + |  |
| 5720  | 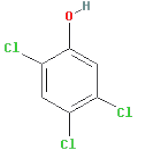   | + |  |
| 5721  | 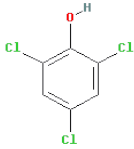 | + |  |
| 11    | 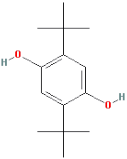 | - |  |
| 166   | 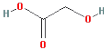 | - |  |

|      |                                                                                     |   |  |
|------|-------------------------------------------------------------------------------------|---|--|
| 180  | 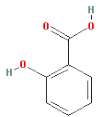   | - |  |
| 774  | 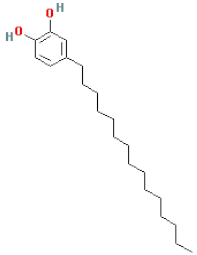   | - |  |
| 776  | 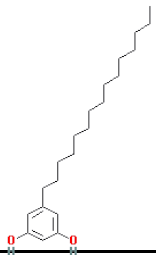   | - |  |
| 1047 | 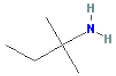 | - |  |
| 1048 | 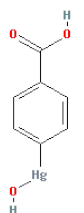 | - |  |
| 1267 | 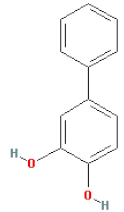 | - |  |

|      |                                                                                     |   |  |
|------|-------------------------------------------------------------------------------------|---|--|
| 1362 | 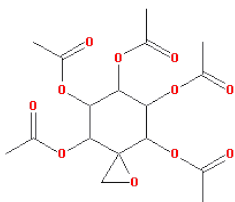   | - |  |
| 1557 | 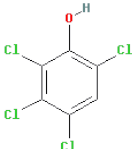   | - |  |
| 1566 | 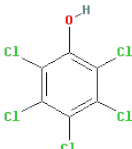   | - |  |
| 1569 | 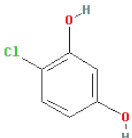 | - |  |
| 1570 | 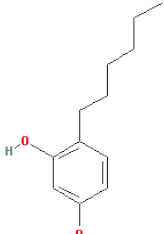 | - |  |
| 1571 | 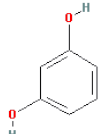 | - |  |

|      |                                                                                     |   |  |
|------|-------------------------------------------------------------------------------------|---|--|
| 1572 | 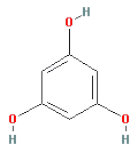   | - |  |
| 1573 | 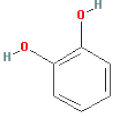   | - |  |
| 1577 | 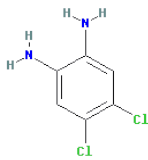   | - |  |
| 1696 | 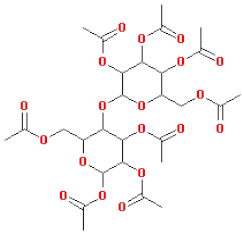 | - |  |
| 1839 | 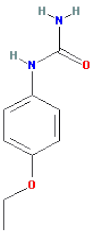 | - |  |
| 1857 | 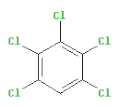 | - |  |

|      |                                                                                     |   |  |
|------|-------------------------------------------------------------------------------------|---|--|
| 2165 | 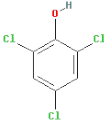   | - |  |
| 2266 | 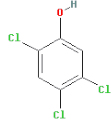   | - |  |
| 2383 | 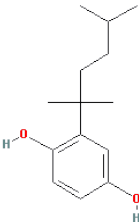   | - |  |
| 2428 | 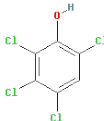 | - |  |
| 2818 | 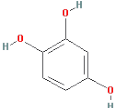 | - |  |
| 2879 | 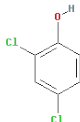 | - |  |

|      |                                                                                     |   |  |
|------|-------------------------------------------------------------------------------------|---|--|
| 3759 | 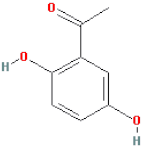   | - |  |
| 3770 | 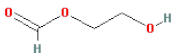   | - |  |
| 3949 | 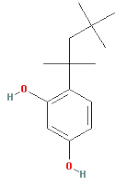   | - |  |
| 4389 | 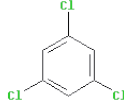 | - |  |
| 4763 | 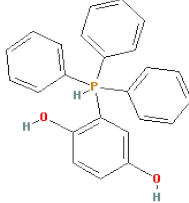 | - |  |
| 4962 | 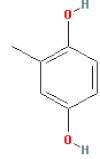 | - |  |

|      |                                                                                     |   |  |
|------|-------------------------------------------------------------------------------------|---|--|
| 4972 | 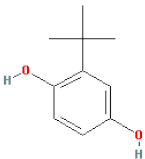   | - |  |
| 5035 | 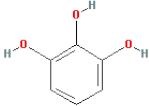   | - |  |
| 5310 | 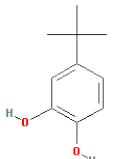   | - |  |
| 6298 | 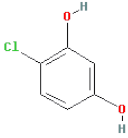 | - |  |
| 7930 | 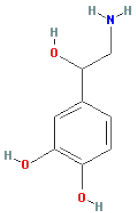 | - |  |
| 8643 | 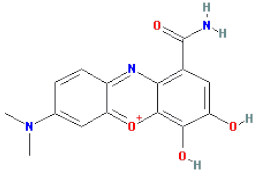 | - |  |

|      |                                                                                     |   |  |
|------|-------------------------------------------------------------------------------------|---|--|
| 8690 | 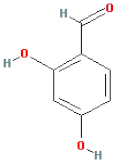   | - |  |
| 9177 | 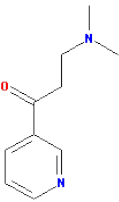   | - |  |
| 9243 | 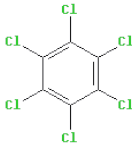   | - |  |
| 9247 | 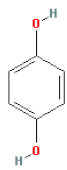 | - |  |
| 9788 | 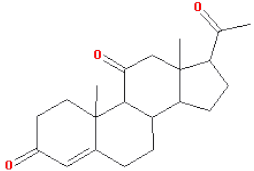 | - |  |
| 9879 | 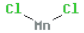 | - |  |

|       |                                                                                     |   |  |
|-------|-------------------------------------------------------------------------------------|---|--|
| 9924  | 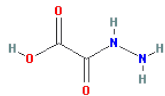   | - |  |
| 10785 | 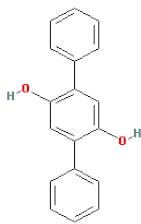   | - |  |
| 11607 | 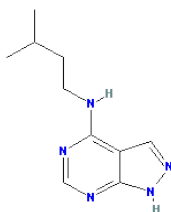   | - |  |
| 13018 | 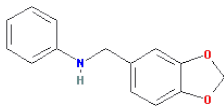 | - |  |
| 14422 | 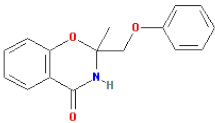 | - |  |
| 14872 | 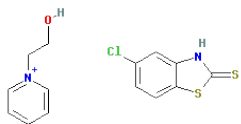 | - |  |

|       |                                                                                     |   |  |
|-------|-------------------------------------------------------------------------------------|---|--|
| 15317 | 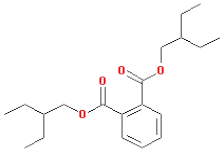   | - |  |
| 16149 | 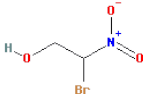   | - |  |
| 16155 | 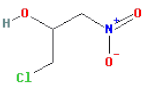   | - |  |
| 16228 | 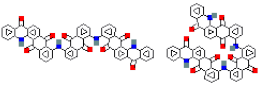 | - |  |
| 21235 | 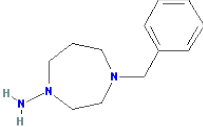 | - |  |
| 22961 | 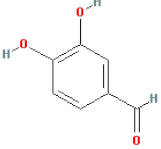 | - |  |

|       |                                                                                     |   |  |
|-------|-------------------------------------------------------------------------------------|---|--|
| 23528 | 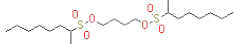   | - |  |
| 25316 | 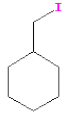   | - |  |
| 27003 | 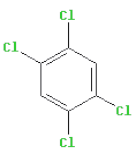   | - |  |
| 28994 | 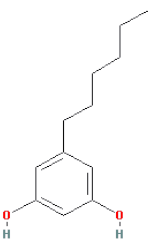  | - |  |
| 29088 | 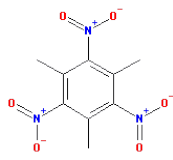 | - |  |
| 29976 | 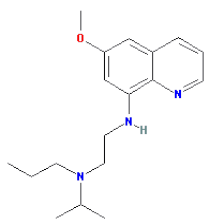 | - |  |

|       |                                                                                     |   |  |
|-------|-------------------------------------------------------------------------------------|---|--|
| 34645 | 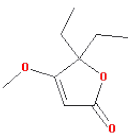   | - |  |
| 34803 | 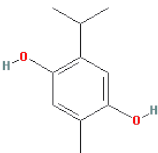   | - |  |
| 35959 | 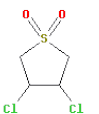   | - |  |
| 36955 | 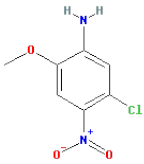 | - |  |
| 36978 | 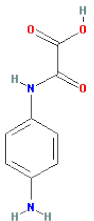 | - |  |
| 39877 | 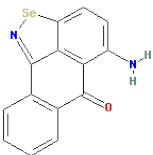 | - |  |

|       |                                                                                     |   |  |
|-------|-------------------------------------------------------------------------------------|---|--|
| 40737 | 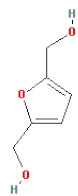   | - |  |
| 40844 | 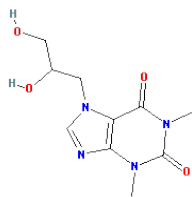   | - |  |
| 43118 | 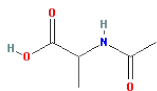   | - |  |
| 43432 | 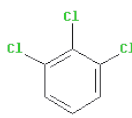 | - |  |
| 43554 | 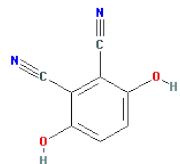 | - |  |
| 43641 | 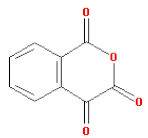 | - |  |

|       |                                                                                     |   |  |
|-------|-------------------------------------------------------------------------------------|---|--|
| 46511 | 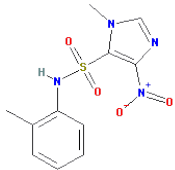   | - |  |
| 47883 | 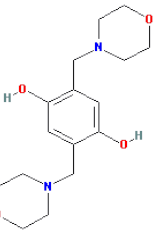   | - |  |
| 49356 | 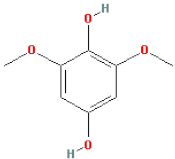   | - |  |
| 50729 | 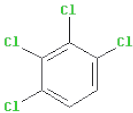 | - |  |
| 55746 | 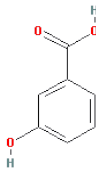 | - |  |
| 57752 | 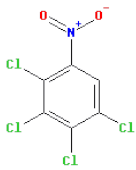 | - |  |

|       |                                                                                     |   |  |
|-------|-------------------------------------------------------------------------------------|---|--|
| 60646 | 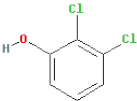   | - |  |
| 60647 | 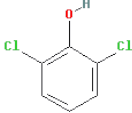   | - |  |
| 60648 | 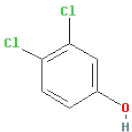   | - |  |
| 60649 | 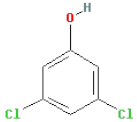 | - |  |
| 60650 | 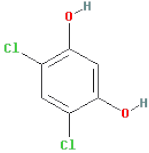 | - |  |
| 62687 | 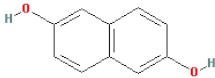 | - |  |

|        |                                                                                     |   |  |
|--------|-------------------------------------------------------------------------------------|---|--|
| 78934  | 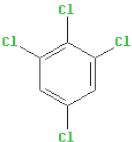   | - |  |
| 79456  | 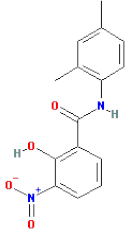   | - |  |
| 82029  | 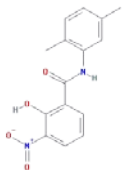   | - |  |
| 99308  | 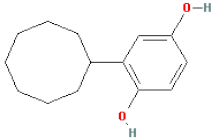 | - |  |
| 108080 | 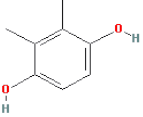 | - |  |
| 144257 | 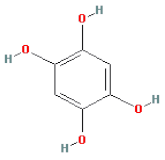 | - |  |

|        |                                                                                     |   |  |
|--------|-------------------------------------------------------------------------------------|---|--|
| 269124 | 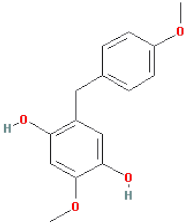   | - |  |
| 281309 | 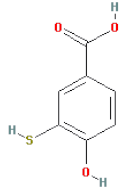   | - |  |
| 400633 | 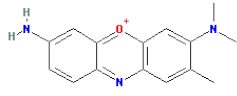   | - |  |
| 401617 | 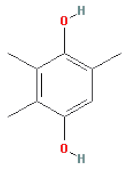 | - |  |
| 401619 | 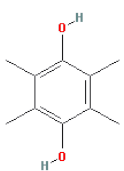 | - |  |
| 406697 | 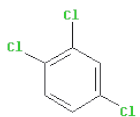 | - |  |

|        |                                                                                     |   |  |
|--------|-------------------------------------------------------------------------------------|---|--|
| 407823 | 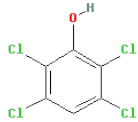   | - |  |
| 528169 | 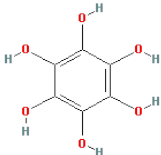   | - |  |
| 604580 | 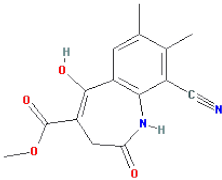   | - |  |
| 635971 | 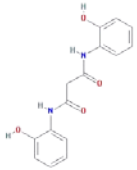 | - |  |

Supplement: Additional file 1 — Table S1. TRABID inhibitory activity and structures of additional compounds. [file 1472-6769-12-4-S1.pdf]
